# Effect of glycosphingolipids on osteoclastogenesis and osteolytic bone diseases

**DOI:** 10.3389/fendo.2012.00106

**Published:** 2012-08-23

**Authors:** Adel Ersek, Anastasios Karadimitris, Nicole J. Horwood

**Affiliations:** ^1^Kennedy Institute of Rheumatology, Nuffield Department of Orthopaedics, Rheumatology and Musculoskeletal Sciences, University of OxfordLondon, UK; ^2^Centre for Haematology, Department of Medicine, Imperial College London, Hammersmith HospitalLondon, UK

**Keywords:** glycosphingolipids, osteoclast, Gaucher’s disease, multiple myeloma, lipid raft

## Abstract

Alterations in glycosphingolipid (GSL) production results in lysosomal storage disorders associated with neurodegenerative changes. In Gaucher’s disease, the patients also develop osteoporosis that is ameliorated upon treatment for the underlying defect in GSL metabolism. The role of GSLs in osteoclast and osteoblast formation is discussed here as well as the potential therapeutic uses of already approved drugs that limit GSL production in bone loss disorders such as multiple myeloma and periodontal disease.

## INTRODUCTION

Endocrine disorders such as Cushing’s syndrome and hyperthyroidism are well known for their deleterious effects on bone density (Walker-Bone, 2012). Disorders with chronic inflammation such as rheumatoid arthritis and inflammatory bowel disease also present with systemic bone loss (Miazgowski et al., 2012). However, the importance of osteoporosis in some patients can be overlooked due to more immediate health concerns. It may be seen as a secondary complication attributed to treatment regime, such as long-term glucocorticoid use, whereas it may in fact be telling us more about a direct effect of the primary pathology on bone homeostasis. In lysosomal storage disorders (LSDs), such as Gaucher’s disease, patients present with osteoporotic bone loss, in addition to characteristic hematological and neurological defects, that improves with treatment for the underlying defect in glycosphingolipid (GSL) metabolism. Clinical observations show that enzyme replacement therapy (ERT) affects favorably not just the nervous system but also ameliorates the reduction in bone mineral density and consequent osteoporosis (El-Beshlawy et al., 2006; Pastores et al., 2007).

## WHAT ARE GLYCOSPHINGOLIPIDS?

Sphingolipids and their glycosylated derivatives, GSLs constitute a diverse array of lipids in which a ceramide lipid backbone is linked to one or more saccharides (Fyrst and Saba, 2010; Kolter, 2011).

**FIGURE 1:**
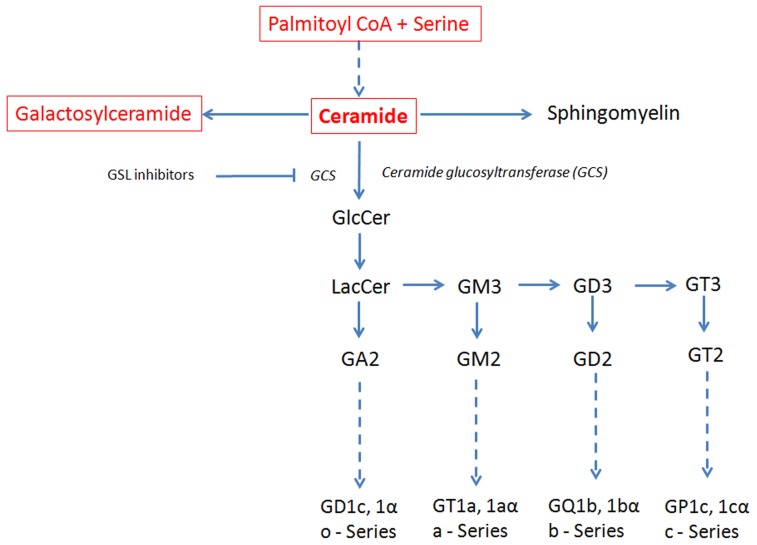
**Schematic view of the GSL *de novo* biosynthesis pathway.** Ceramide, the metabolic precursor of complex sphingolipids, occupies a central position in the GSL biosynthesis. Ceramide synthesis occurs in the ER, while at the level of *cis*-Golgi apparatus GlcCer is generated that is converted in LacCer in the *trans*-Golgi. The reactions involving ceramide transformation into all major classes of GSL are catalyzed in the lumen of the Golgi apparatus by membrane-bound transferases. Ceramide glucosyltransferase (GCT), also known as glucosylceramide synthase (GCS), is the rate limiting step for the synthesis of all major classes of GSL. GSL inhibitors like NB-DNJ inhibit GCS and hence the biosynthesis of all major GSL. Dashed line: intermediate synthesis steps are not shown. Sphingolipids synthesized in the ER are in red and in a box; GSL synthesized in the Golgi are in black. The GSL synthesis rate limiting enzyme GCS is shown in italics.

Sphingolipids contain a sphingoid base (sphingosine in mammalian cells) that by acylation with a fatty acid results in a ceramide moiety as their core; the addition of a phosphocholine head group generates sphingomyelin, while the addition of sugars to the ceramide moiety generates GSLs (**Figure 1**). When the head group contains the negatively charged sugar sialic acid, the GSL are referred to as gangliosides, whereas when they lack sialic acid, they are called neutral GSL. The complex processes of biosynthesis and degradation of sphingolipids and GSL involve numerous enzymes located in various subcellular compartments. The *de novo* biosynthesis of GSLs is initiated at the cytoplasmic face of the endoplasmic reticulum (ER) by serine and palmitoyl-CoA condensation that ultimately generates ceramide. Conversion of ceramide to glucosylceramide (GlcCer) by glucosylceramide synthase (GCS) is a critical and rate limiting biochemical step in GSL biosynthesis (Ichikawa and Hirabayashi, 1998; Fyrst and Saba, 2010; Xu et al., 2010). Subsequently, GlcCer is converted to lactosylceramide (LacCer) by β-(1,4) transfer of galactose from UDP-galactose by galactosyltransferase I (Nomura et al., 1998). LacCer provides the common substrate for the synthesis of more complex GSL (Huwiler et al., 2000). The stepwise conversion of LacCer to the mono-, di-, and trisialo-gangliosides (GM3, GD3, and GT3, respectively) involving the sequential activities of sialyltransferases and glycosyltransferases in turn gives rise to the precursors for the synthesis of the o-, a-, b-, and c-series of GSL with none, one, two, or three sialic residues attached to the 3-position of the galactose residue of LacCer (Lahiri and Futerman, 2007; Fuller, 2010; **Figure 1**). After their biosynthesis, GSLs are transported by exocytosis to the plasma membrane in which they integrate. Besides *de novo* biosynthesis, GSL can also be formed by metabolic recycling of the building blocks (such as monosaccharides, sphingosine, and ceramide) released in their catabolic degradation. This recycling of catabolic degradation products for biosynthetic purposes is known as metabolic salvage pathway (Tettamanti, 2003; Tettamanti et al., 2003).

Constitutive degradation of GSLs takes place in endosomes and lysosomes. Together with other membrane components, GSLs enter the acidic compartment via endocytosis and their terminal carbohydrate residues are sequentially cleaved off by lysosomal glucosidases obtaining ceramide which can then leave the lysosome to be recycled together with other cleavage products within the mentioned salvage pathways or be further degraded (Sandhoff and Kolter, 2003; Kolter, 2011). In the non-lysosomal degradation process throughout different subcellular localizations, ceramide is subsequently degraded to sphingosine and a fatty acid by the action of a family of ceramidases (Hannun and Obeid, 2002; Sandhoff and Kolter, 2003; Kolter and Sandhoff, 2005).

Although sphingolipids and GSL are a minor component of the total cellular lipid pool (5–10%), their accumulation in certain cell types forms the basis of many diseases (Butters, 2007a; Kolter, 2011). Biosynthesis and degradation of these lipids is closely regulated by numerous enzymes, and the failure of a given enzyme to participate in the metabolism results in the accumulation of the enzyme’s substrate, giving rise to lysosomal storage diseases. Defective GSL catabolism and degradation characterizes a group of LSD called the glycosphingolipidoses (see below; Butters, 2007b; Fuller, 2010; Cox and Cachon-Gonzalez, 2012).

### GSL IN PHYSIOLOGY AND DISEASE

Glycosphingolipid, constituents primarily of the outer leaflet of the cellular plasma membrane, vary between different tissues and during cell differentiation. This variability reflects their differing functional roles in many cellular processes including modification of cell signaling initiated by tyrosine kinases at the cell membrane, cell cycle control and apoptosis, adhesion, and migration (Kolter and Sandhoff, 2006). While, at the single-cell level and in *in vitro* cultures, GSL are not essential for cell survival or even differentiation, embryonic lethality of GCS knockout mice suggests that they are critical for cellular processes at the multicellular whole-organism level (Yamashita et al., 1999).

Glycosphingolipidoses are rare autosomal recessive disorders characterized by defects in GSL catabolism and accumulation of GSL substrates in lysosomes. These include GM1-gangliosidosis, GM2-gangliosidosis, Tay-Sachs, Krabbe, Fabry, Sandhoff, Farber, Niemann–Pick, and three subtypes of Gaucher’s disease (Kolter and Sandhoff, 2006) with type I being by far the most common (Hughes et al., 2007). Gaucher’s disease is caused by partial or severe deficiency of lysosomal β-glucocerebrosidase, resulting in accumulation primarily of *N*-acyl-sphingosyl-1-*O*-β-D-glucoside. In type I Gaucher’s disease, partial deficiency of glucocerebrosidase is associated with the accumulation of GSL in macrophages resulting in a distinctive morphology (Gaucher’s cells). These cells are present in the liver, bone marrow, and spleen leading to the clinical manifestations of massive hepatosplenomegaly and bone marrow failure with peripheral blood cytopenias. Type II and III Gaucher’s disease are additionally characterized by variable neurological abnormalities and an overall worse prognosis than type I. Furthermore, patients with Gaucher’s disease develop progressive osteoporosis and often osteolytic lesions, believed to reflect increased osteoclastic activity.

In addition to ERT, a useful and effective treatment option for Gaucher’s disease and other glycosphingolipidoses is substrate reduction therapy (SRT). This is aimed at depleting GSL biosynthesis via inhibition of key enzyme ceramide-specific glucosyltransferase (**Figure 1**) by *N*-alkylated imino sugars, e.g., *N*-butyldeoxynojirimycin (NB-DNJ; Miglustat) or *N*-butylde-oxygalactonojirimycin (NB-DGJ; Butters et al., 2005). However, the glucose analog, NB-DNJ, is also an inhibitor of intestinal and ER α-glucosidases, and can lead to bowel irritation in man.

In Gaucher’s disease, the infiltration of the bone marrow by glucocerebroside-laden macrophages triggers a diverse pattern of skeletal disease that results in crippling complications, including osteoporosis. The severity and pattern of skeletal disease determines disease status and the response to SRT (Butters, 2007a). Notably, NB-DNJ has been shown to significantly improve osteoporosis (bone mineral density) and associated clinical manifestations (e.g., bone pain, risk of a vascular necrosis, and fractures) in patients with Gaucher’s disease (El-Beshlawy et al., 2006; Pastores et al., 2007). This salutary effect is probably linked to the relatively selective uptake of NB-DNJ by cells of the macrophage lineage to which the osteoclast belongs. Bone involvement in other glycosphingolipidoses is less well described. In an animal model of Krabbe disease, the femora of twitcher mice are smaller than of those of wild type mice, and present with abnormality of marrow cellularity, bone deposition (osteoblastic function), and osteoclastic activity (Contreras et al., 2010). A mouse model emulating type I Gaucher’s disease has been created by the conditional deletion of the glucocerebrosidase gene (GBA1) in the hematopoietic and mesenchymal cell lineages using the Mx1 promoter. Analysis of these mouse revealed that they fully recapitulated the human disease in terms of cytokine measurements, microarray analysis, and cellular immunophenotyping. Additionally, there was widespread dysfunction of macrophages, thymic T cells, dendritic cells, and osteoblasts leading to a dramatic loss of bone architecture (Mistry et al., 2010).

In cancers, alterations in the cellular GSL profile have long been recognized as a trait of malignant transformation (Hakomori, 2001). Surface-bound as well as shed GSL have been shown to modulate cellular functions that promote tumor survival and growth, metastasis, and angiogenesis (Hakomori, 2001; Birkle et al., 2003). In acute myeloid leukemia (AML) patients there is higher expression of the GSL lactotriaosylceramide (Lc3), GM3, and neolactotetraosylceramide (nLc4) in the bone marrow compared to healthy donors. It is thought that these GSL may be involved in the initiation and differentiation of AML (Wang et al., 2012). Oncogene-transformed fibroblasts from GM3 synthase/GM2 synthase double knockout mice are not able to form complex gangliosides and displayed significantly impaired tumor growth in syngeneic immunocompetent mice, underscoring the pivotal role of tumor cell-derived gangliosides (Liu et al., 2010).

### GLYCOSPHINGOLIPIDS AND LIPID RAFT FORMATION

Together with glycerophospholipids and cholesterol, GSL are building blocks of eukaryotic membranes. On the surface of mammalian cells GSL are not homogenously distributed but form patterns that are characteristic of the cell type, and alter in response to cell growth, differentiation, oncogenesis, and external stimuli. Along with sphingomyelin and cholesterol, GSL form membrane microdomains known as lipid rafts (Simons and Ikonen, 1997; Pike, 2006). These are transient dynamic, detergent-insoluble structures (Eggeling et al., 2009) in which nascent interactions between GSL and different receptor proteins are essential for initiating a variety of signaling cascades.

Membrane lipid rafts play a key role in immune cell activation by recruiting and excluding specific signaling components of immune cell surface receptors upon receptor engagement. Signaling through lipid raft associated GSL activity was shown to be important in many cell types including osteoclasts (Ha et al., 2003b). The ganglioside GM1, also known as a GSL-enriched microdomain (GEM) marker, is invariably associated with these structures. Of the hundreds of different GSL species present in biological membranes, lipid raft biophysical studies have mainly been performed with only a few representative species. These include, e.g., gangliosides GM3 and GM1, LacCer, sulfatides, and the small neutral monoglycosylceramides (GlcCer and GalCer; Westerlund and Slotte, 2009). Studies indicate that variations in the particular GSLs present in lipid rafts determines cellular signaling capacity (Pike, 2006).

### GLYCOSPHINGOLIPIDS, LIPID RAFTS, AND OSTEOCLASTOGENESIS

Osteoclasts, the bone resorbing cells, form by the fusion of mononuclear precursors in the presence of two major pro-osteoclastogenic cytokines, receptor activator of NF-κB ligand (RANKL), a surface-bound or soluble cytokine, and macrophage-colony stimulating factor (M-CSF; Horwood et al., 1998; Hsu et al., 1999; Kong et al., 1999). Osteoclast formation and activation can be further enhanced via the combination of these factors with inflammatory cytokines and growth factors present in disease (Horwood, 2008). The role of GSLs in osteoclast development and activity is an area of on-going research.

Previous work has shown that LacCer, GM2, and GM3 are the main GSL constituents of mature osteoclasts (Iwamoto et al., 2001) while GM1 co-localizes with RANK, the RANKL receptor, in lipid rafts (Ha et al., 2003b). Inhibition of GSL synthesis by the non-specific GCS inhibitor D-threo-1-phenyl-2-decanoylamino-3-morpholino-1-propanol (D-PDMP; Iwamoto et al., 2001) or chemical disruption of lipid rafts prevents RANKL driven osteoclast development (Ha et al., 2003b): the expression of RANK was reduced markedly in D-PDMP-treated cells. An *in vitro* synergistic effect of exogenous LacCer in RANKL-dependent osteoclastogenesis was also shown. Exogenous GM3 and GM1 was able to restore osteoclast formation but to a lesser extent than LacCer (Iwamoto et al., 2001). Likewise, we have shown that GM3 is a pro-osteoclastogenic factor that synergistically enhances the ability of the pro-osteoclastogenic factors RANKL and insulin-like growth factor-1 (IGF-1) to induce the maturation of osteoclasts. Inhibition of GSL synthesis using the imino sugar NB-DNJ, a more specific GCS inhibitor, dramatically inhibited RANKL-induced osteoclastogenesis (Xu et al., 2009).

Lipid rafts are essential for osteoclast development and activation as shown by the finding that cholesterol depletion by methyl-β-cyclodextrin impairs the ruffled border-targeted vesicle trafficking pathway and bone resorption (Mulari et al., 2008). Consistent with a role of rafts in osteoclast activation, the raft component flotillin greatly increased during osteoclast differentiation. Recent investigations oriented toward elucidating the effects of GSL synthesis inhibitors in osteoclast development and function proposed that these compounds are able to regulate osteoclastogenesis by interfering with RANK, c-Src and TRAF6 co-localization in the lipid raft, thereby ultimately interfering with the signaling cascade that activates the NF-κB pathway and the subsequent transcription of osteoclastogenic genes (Ha et al., 2003a; Fukumoto et al., 2006). Proximal signaling events following engagement of RANK by RANKL includes translocation of TRAF6 to rafts where Src is constitutively resident. Disruption of rafts by GSL inhibitors blocked TRAF6 translocation and Akt activation in response RANKL and further reduced the survival and actin ring formation of osteoclasts (Ha et al., 2003b).

Bahtiar et al. (2009) identified the amino acid L-Ser in the differentiation medium as necessary for the expression of NFAT2, a transcription factor critical for osteoclast activation and function. Serine analogs that antagonize the function of L-Ser suppressed the formation of osteoclasts in bone marrow as well as the expression and localization of RANK in membrane lipid rafts; the addition of LacCer rescued the osteoclastic formation. When administered *in vivo*, the analog significantly increased bone density in mice and prevented high bone turnover induced by treatment with soluble RANKL (Bahtiar et al., 2009). The impact on other GSLs was not investigated but given our findings and those of others it is likely that GM3 would also be able to rescue osteoclast activity. Since L-Ser is required for formation of ceramide and ultimately of GSL (see **Figure 1**), an interesting possibility, compatible with the ability of LacCer to rescue the inhibitory effect of Ser analogs, is that the pro-osteoclastogenic effect of L-Ser is mediated through increased biosynthesis and presence of GSL in lipid rafts, promoting RANKL-dependent signaling.

### GLYCOSPHINGOLIPIDS AND OSTEOBLAST DEVELOPMENT

Much less is known regarding the role of GSL on the development of osteoblasts, the bone forming cells that in concert with osteoclasts are responsible for bone remodeling and homeostasis. Inhibition of ST3 β-galactoside α-2, 3-sialyltransferase 2 (ST3Gal II), the enzyme required for ganglioside GD1a synthesis (**Figure 1**) resulted in reduced osteoblast differentiation as measured by alkaline phosphatase levels. This was due to reduced phosphorylation of extracellular signal-regulated kinases (ERK) 1/2 mitogen-activated protein (MAP) kinase and epidermal growth factor receptor (EGFR; Yang et al., 2011). High-performance thin-layer chromatography of human mesenchymal stem cells (MSCs) showed that ganglioside GM3 expression was decreased, whereas ganglioside GD1a expression was increased during the differentiation of MSCs into osteoblasts. Furthermore, treatment with GM3 reduced alkaline phosphatase production by osteoblasts and reduced EGFR phosphorylation (Kim et al., 2008). More recently, in the GBA1 conditional deletion mice, a defect in osteoblast activity *in vitro* and *in vivo* was discovered. This resulted in severe osteoporosis due to a defect in osteoblastic bone formation arising from an inhibitory effect of the accumulated lipids (LysoGL-1 and GL-1) on protein kinase C activity (Mistry et al., 2010).

Taken together with the effects of GM3 on osteoclastogenesis, this would suggest that increasing levels of GM3, as seen in multiple myeloma (MM) patients (see below), would be indicative of bone loss due to excessive osteoclast activity and a failure of osteoblast activity. Further investigation of other bone loss disorders and their GSL profiles remains to be completed.

## THERAPEUTIC POSSIBILITIES TARGETING GLYCOSPHINGOLIPID PRODUCTION IN BONE DISEASES

### BONE DISEASE IN GAUCHER’S DISEASE

While ERT and SRT clearly benefit on bone disease and osteoporosis in patients with Gaucher’s disease, the exact cellular and molecular basis of bone disease in these patients are not fully understood.

Increased levels of cathepsin K, the cysteine protease secreted by activated osteoclasts and responsible for organic bone degradation, have been reported in the serum and spleens of patients with Gaucher’s disease (Moran et al., 2000). Whether this reflects increased frequency and activity of osteoclasts has not been studied directly either in patients or animal models of Gaucher’s disease. In addition, the pro-inflammatory milieu associated with Gaucher’s disease, and in particular the elevated levels of the pre-osteoclastogenic cytokines interleukin (IL)-1, IL-6, and tumor necrosis factor-α (TNF-α) secreted by pathological macrophages, might be another important parameter in the pathogenesis of bone disease in these patients (de Fost et al., 2008).

Given the tightly regulated cross-talk between osteoclasts and osteoblasts, osteoblast function is likely to be altered in Gaucher’s disease. This has been confirmed in the GBA1 conditional deletion mice (Mistry et al., 2010). Although direct evidence of this is lacking in humans, analysis of the cellular biochemistry of MSCs from an adult patient with Gaucher’s disease type I (N370S/L444P mutations), showed that Gaucher’s MSCs have a marked increase in COX-2, prostaglandin E_2_, IL-8, and CCL2 production compared with normal controls (Campeau et al., 2009). Additionally, Lecourt et al. (2012) have used an *in vitro* chemical model of GBA depletion with Conduritol B Epoxide (CBE), a specific inhibitor of GBA activity, to assess capacity of bone marrow MSC to differentiate in to osteoblasts. They observed a dramatic impairment of MSCs proliferation and although the capacity of MSCs to differentiate into osteoblasts was not altered, the expression of IL-6, IL-8, monocyte chemoattractant protein-1 (MCP1), dickkopf-1 (DKK1), and stromal cell-derived factor 1 (SDF1) were all increased. Furthermore, conditioned media from CBE-treated MSCs enhanced osteoclastic bone resorption (Lecourt et al., 2012). The expression of RANKL and OPG by these cells has not been investigated however it is likely given the increase in inflammatory cytokines that these osteoclast promoting factors will be elevated and could contribute to skeletal disease and immune disease manifestations in a manner distinct and additive to Gaucher’s macrophages themselves.

### BONE DISEASE IN MULTIPLE MYELOMA

The hematological malignancy, MM, is associated with osteolytic bone lesions and skeletal complications in over 80% of patients (Roodman, 2010). Interactions between myeloma cells and cells of the bone marrow microenvironment promote both tumor growth and survival and bone destruction, and the osteolytic bone disease is now recognized as a contributing component to tumor progression. Since myeloma bone disease is associated with both an increase in osteoclastic bone resorption and a suppression of osteoblastic bone formation, research to date has largely focused upon these cells. However, it is now clear that other cell types within the bone marrow, including cells of the immune system, MSCs and bone marrow stromal cells, can contribute to the development of myeloma bone disease (Fowler et al., 2011).

Osteolytic lesions are localized to areas adjacent to tumor growth and are characterized by increased activity of osteoclasts and suppression of osteoblastogenesis (Roodman, 2010). Direct interaction of MM plasma cells with bone marrow stromal cells and osteoblasts is the trigger for the production of a number of cytokines, such as IL-6, that act in an auto- or paracrine fashion to promote survival and growth of the tumor itself and increase osteoclast activity resulting in bone loss (Podar et al., 2009). The activation of osteoclasts in MM is thought to occur in response to osteoclast activating factors including RANKL and IGF-1. Despite different current clinical strategies to stop osteoclast activation that include myeloma-specific therapy such as bortezomib, or treatment specific to osteoclasts such as bisphosphonates, bone disease remains a serious clinical problem (Zangari et al., 2012).

Earlier work on the glycolipid profile of myeloma cell lines showed increased expression of gangliosides GM2 and GM3 and of the neutral GSL LacCer and globosides Gb3 and Gb4 (Kalisiak et al., 1991; O’Boyle et al., 1996). This combination of increased production of specific GSL along with osteoclastogenic cytokine production would lead to dramatically enhance osteoclast formation and consequent bone destruction. It is possible that inhibition of *de novo* GSL synthesis in osteoclasts by *N*-alkylated imino sugars like Miglustat would reduce both osteoclast formation and tumor growth, either alone or in combination therapy with the proteasome inhibitor, bortezomib, and other established anti-myeloma agents. This combination would thus treat both the bone disease and the problem of MM cells failing to undergo appropriate apoptosis.

An interesting link between myeloma and Gaucher’s disease exists: patients with Gaucher’s disease have a 6–50 times increased risk of developing myeloma as well as of the pre-myeloma condition monoclonal gammopathy of uncertain significance (Rosenbloom et al., 2009). Although increased secretion of IL-6, a cytokine critical for myeloma survival, by the pathological Gaucher’s disease macrophages might play an important role, the complete pathogenetic basis of increased risk of myeloma in Gaucher’s disease remains to be elucidated. Nevertheless, this unique relationship between Gaucher’s disease and myeloma further underscores the role of GSL in osteoclastogenesis and the potential of imino sugar inhibitors as treatment for osteoclastic bone disease.

### BONE LOSS IN PERIODONTAL DISEASE

Lipopolysaccharide (LPS) and lipid A, lipoprotein, fimbriae, and phosphorylated dihydroceramides of *Porphyromonas gingivalis* have been reported to lead to osteoclast modulation and alveolar bone loss via TLR2 interaction. Both the LPS and lipid A derived from *P. gingivalis* are contaminated with phosphorylated dihydroceramide lipids and the proportion of these lipids increases with disease suggesting that TLR2 activation of host tissues attributed to LPS and lipid A of *P. gingivalis* could actually be mediated by phosphorylated dihydroceramides (Nichols et al., 2012). Furthermore, *P. gingivalis* lipids have been reported to inhibit osteoblast function and gene expression (Wang et al., 2010). Conversely, ceramide signaling has been reported to stimulate osteoblast survival and apoptosis; this effect of ceramide on cell viability was specific as C(2)-dihydroceramide had no effect. The authors propose that alteration in the intracellular levels of ceramide may be important in bone remodeling (Hill and Tumber, 2010). Thus in periodontal disease, the elevated levels of phosphorylated dihydroceramides will promote osteoclast function whilst inhibiting osteoblast function leading to net bone loss. Using patients with treatment resistant periapical lesions, the relative proportions of GSL have been determined showing an increase in the presence of GM3 (Zuolo et al., 2001). Thus alteration in GSL synthesis and the presence of bacterially derived lipids leads to the activation of osteoclasts and the inhibition of osteoblasts. In combination with elevated inflammatory cytokine production this will lead to the consequent bone depletion and tooth loss.

## FUTURE DIRECTIONS

The characterization of GSLs in bone tumors, such as giant cell tumor of bone and the various osteosarcomas, remains to be described. The reversal of osteoporosis in Gaucher’s patients following treatment for their LSD combined with the direct effects of ganglioside GM3 on osteoclastogenesis and the alterations in GSLs observed in other diseases suggests that targeting GSLs may improve osteoclastic bone loss. The effectiveness of targeting GSL in patients with osteoporosis that is not a result of LSD requires further investigation. Likewise the role of GSLs in osteoblast formation and activation is an exciting area for future bone anabolic therapies.

The sheer number and diversity of GSLs and related molecules makes this field of research an extremely challenging one. However, all the signs are showing that modifying the expression levels of GSLs may be therapeutically beneficial, not just for patients with Gaucher’s disease but also for cancer and inflammation-associated bone loss. The fact that imino sugar inhibitors, such as Miglustat (NB-DNJ), have been used therapeutically in patients with Gaucher’s disease (Butters, 2007b) provides an approved drug along with safety profile information that would allow swift application of these inhibitors to bone loss diseases.

## Conflict of Interest Statement

The authors declare that the research was conducted in the absence of any commercial or financial relationships that could be construed as a potential conflict of interest.
